# Comparison between physical and cognitive treatment in patients with MIC and Alzheimer’s disease

**DOI:** 10.18632/aging.101970

**Published:** 2019-05-24

**Authors:** Cristina Fonte, Nicola Smania, Anna Pedrinolla, Daniele Munari, Marialuisa Gandolfi, Alessandro Picelli, Valentina Varalta, Maria V. Benetti, Annalisa Brugnera, Angela Federico, Ettore Muti, Stefano Tamburin, Federico Schena, Massimo Venturelli

**Affiliations:** 1 Neuromotor and Cognitive Rehabilitation Research Centre, Department of Neurosciences, Biomedicine and Movement Sciences, University of Verona, Verona, Italy; 2 Neurorehabilitation Unit, Department of Neurosciences, Hospital Trust of Verona, Verona, Italy; 3 Department of Neurosciences, Biomedicine and Movement Sciences, University of Verona, Verona, Italy; 4 Mons. A. Mazzali Foundation, Mantua, Italy; 5 Department of Internal Medicine, University of Utah, Salt Lake City, UT 84112, USA

**Keywords:** physical activity, cognitive therapy, dementia, Alzheimer’s disease, mini-mental state examination

## Abstract

Cognitive and physical activity treatments (CT and PT) are two non-pharmacological approaches frequently used in patients with Mild Cognitive Impairment (MCI) and Alzheimer’s Disease (AD). The aim of this study was to compare CT and PT in these diseases. Eighty-seven patients were randomly assigned to CT (n=30), PT (n=27) or control group (CTRL; n=30) for 6 months. The global cognitive function was measured by Mini Mental State Examination (MMSE). Specific neuropsychological tests explored attention, memory, executive functions, behavioral disorders. Cardiovascular risk factors (CVD) were collected. All measures were performed before (T0), after treatments (T1), and at three-months follow-up (T2). MMSE did not change from T0 to T1 and T2 in patients assigned to PT and CT, while CTRL patients showed a decline MCI: -11.8%, AD: -16.2%). Between group differences (MCI vs AD) were not found at T1 and T2. Significant worsening was found for CTRL in MCI (T0- T1: *P*=.039; T0-T2: *P*<.001) and AD (T0-T1: *P*<.001; T0-T2: *P*<.001), and amelioration was found for CT in AD (T0-T2: *P*<.001). Attention, executive functions and behavioral disorders were unaffected by either PT or CT. Memory was increased in patients with MCI assigned to PT (+6.9%) and CT (+8.5%).. CVD were ameliorated in the PT group. CTRL patients of both groups, revealed significant decline in all functions and no between groups differences were detected. PT appear to ameliorate CVD. Although between groups differences were not found, results suggest a major retention in MCI compared with AD, suggesting that the latter might benefit better of constant rather than periodic treatments. This study confirms the positive effects of CT and PT in mitigating the cognitive decline in MCI and AD patients, and it is the first to demonstrate their similar effectiveness on maintaining cognitive function.

## Introduction

In 2050 the number of people aged ≥60 years will increase by 1.25 billion [1] with an estimate of 115.4 million of persons with dementia [2]. Alzheimer’s disease (AD) is the cause of 60–70% of dementia, affecting 48 million of people worldwide [3], causing severe clinical, social, and economic problems [1].

AD is characterized by intraneuronal fibrillary tangles and extracellular deposit of amyloid plaques (Aβ) coupled with reactive microgliosis, loss of neurons and synapses in the cortex [4]. Deposits of Aβ can lead to cortical dysfunctions resulting in many cognitive impairments such as memory and intellectual disabilities, causing a decline in activities of daily living and interfering with quality of life [5]. Although current pharmacological treatments may improve symptoms, there are no disease-modifying strategies for AD and new non-pharmacological interventions are needed [6].

Individuals with Mild Cognitive Impairment (MCI), which show cognitive changes greater than expected for an individual’s age and education level but do not interfere with daily-life activities, have increased risk of dementia. The estimated global prevalence of MCI is 9.6–21.6% [7,8]. Pharmacological treatments for MCI have modest to no effect, and new therapeutic approaches are needed in this condition [9]. Cognitive stimulation is the most recommended non-pharmacological approach for cognitive symptoms in MCI and mild-to-moderate dementia. Despite these promising results, the evidence for cognitive training is still preliminary [10].

Physical activity treatment (PT) is another non-pharmacological treatment with some efficacy in dementia [11,12]. The potential of PT to attenuate the cognitive decline in healthy elderly is clear [13], but the effects of PT on cognitive decline is less consistent because of methodological limits, such as different exercise interventions and small sample size. A systematic review [14] showed that aerobic and resistance PT had some positive effects on global cognition, executive functions, attention and delayed recall in MCI and no cognitive effects in AD. Other studies indicated that PT improve global cognitive ability and memory in MCI [15]. PT was reported to delay the cognitive decline in persons at risk of or who have AD [12].

Unfortunately, to date these data are still unclear due to the heterogeneity between studies and outcomes [11]. Therefore, further research with additional and more specific neuropsychological measurements are needed. The aim of this study was to compare the effects of cognitive treatment (CT) and PT in older people with AD and in subjects with MCI. Our hypothesis was that both CT and PT would attenuate the progression of cognitive deterioration in AD and MCI_,_ with similar results in primary outcome measure, but different effects in the secondary outcome measures. Specifically, we expected amelioration in the memory domain in CT group, while PT group would exhibit improvements in physical function and attention.

## RESULTS

### Demographic and clinical data

**Figure 1:**
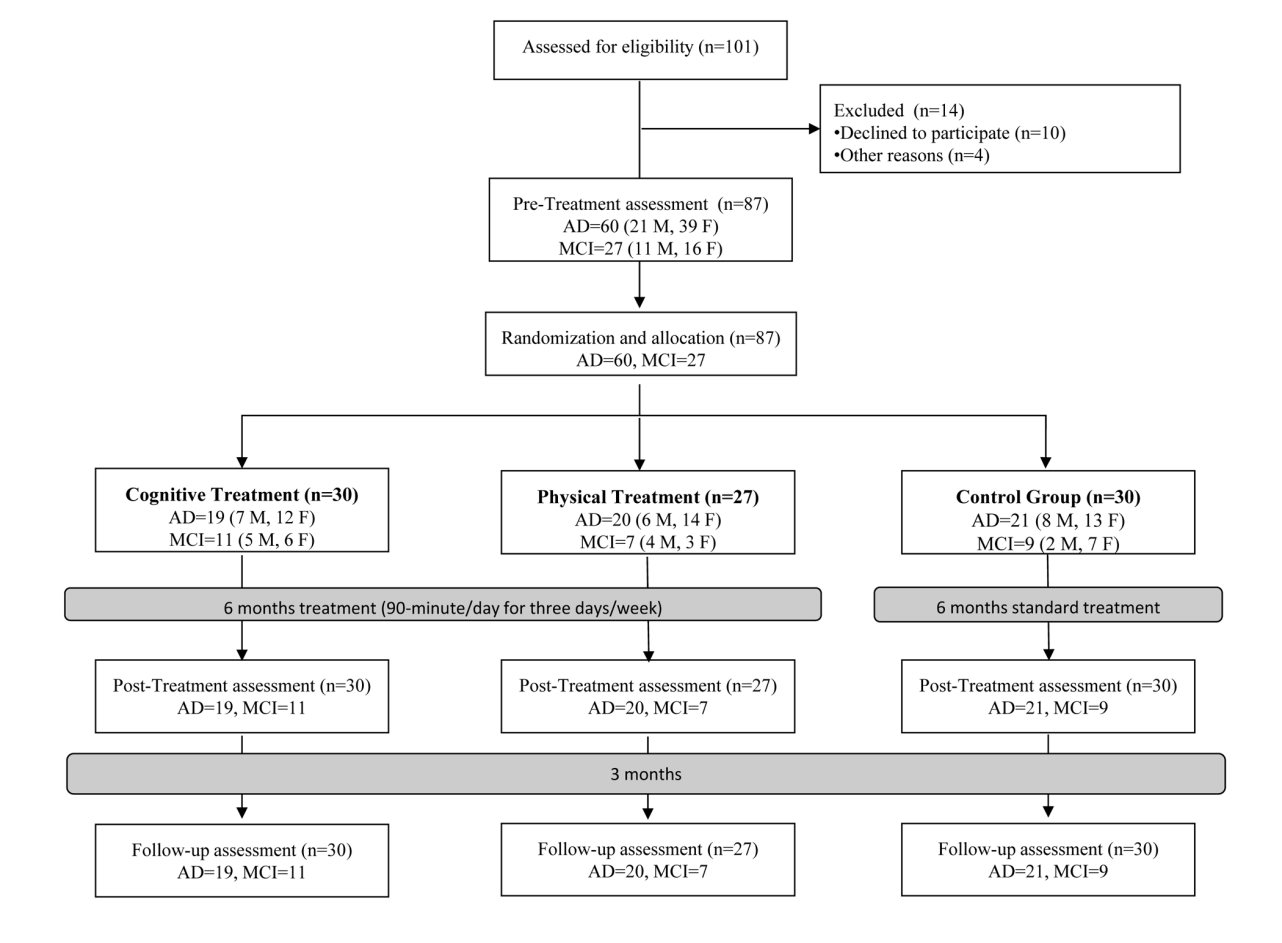
**Flow diagram of the randomized controlled trial.** Abbreviations: MCI: Mild Cognitive Impairment; AD: Alzheimer’s Disease; M: Male; F: Female.

The flow diagram of the study with the specific numbers of participants is reported in Figure 1. The sample was composed of 27 MCI (11 males/16 females) and 60 patients with AD (21 males/39 females). They were randomized to the CT group (n = 30), PT group (n = 27) or the CTRL group (n = 30). Age, education, MMSE and POMA were not statistically different between the three groups of AD and the three groups of MCI at baseline. Patients’ demographic and clinical characteristics are reported in Table 1. Primary and secondary outcomes measures did not significantly differ between the three groups at baseline (T0).

**Table 1:** Demographic data.

		**CT (30)**		**PT (27)**		**CTRL (30)**	
		**AD (19)**	**MCI (11)**	**AD (20)**	**MCI (7)**	**AD (21)**	**MCI (9)**
**Numbers**		7♂/12♀	5♂/6♀	6♂/14♀	4♂/3♀	8♂/13♀	2♂/7♀
**Age (years)**		79±7	76±5	79±9	75±5	80±7	79±3
**Education (years)**		8±5	9±4	7±4	10±4	7±3	8±4
**MMSE (0-30)**		19.6±4.3	26.4±1.4	17.8±5.7	27±2.2	18.7±2.3	25.7±1.8
**POMA (0-28)**		22.9±3.7	25.4±2.3	22.7±2.9	26.1±2.4	23.8±3.2	24.4±3.5
**CDR (0-3)**		9 CDR=1	11 CDR=0.5	9 CDR=1	7 CDR=0.5	11 CDR=1	9 CDR=0.5
		10 CDR=2		11 CDR=2		10 CDR=2	
**Height (m)**		1.65	1.66	1.62	1.67	1.65	1.62
**Weight (kg)**		65.4	73.9	67.4	79.9	67.1	73.0
**Resting HR (bpm)**		40	59	66	59	74	65
**Pharmacological treatment**							
	**Cholinesterase Inhibitors**	9	2	9	1	9	0
	**Antipsychotics**	4	0	5	0	4	0
	**Antidepressants**	8	4	11	3	13	1
	**Benzodiazepines**	2	0	1	0	6	0
**Comorbidity**							
	**Hypertension**	13	8	8	6	11	4
	**Cardiovascular diseases**	10	6	5	2	8	3
	**Diabetes**	1	3	1	3	1	3
	**Arthrosis**	1	1	4	0	1	0

Data are given as mean ± standard deviation. Abbreviations: CT: Cognitive Treatment group; PT: Physical Treatment group; CTRL: Control Group; MMSE: Mini Mental State Examination; POMA: Performance Oriented Mobility Assessment; CDR: Clinical Dementia Rating Scale; Resting HR: Heart Rate at rest.

*= Statistically significant at p ≤ 0.05

### Primary outcomes

Significant effects of the factors Time (F_2,162_= 59.327; *P*<.001), Treatment (F_2,81_= 4.584; *P*=.013) and Group (F_1,81_= 86.707; *P*<.001) and Time X Treatment interaction (F_4,162_= 15.328; *P*<.001) on MMSE were found.

**Figure 2:**
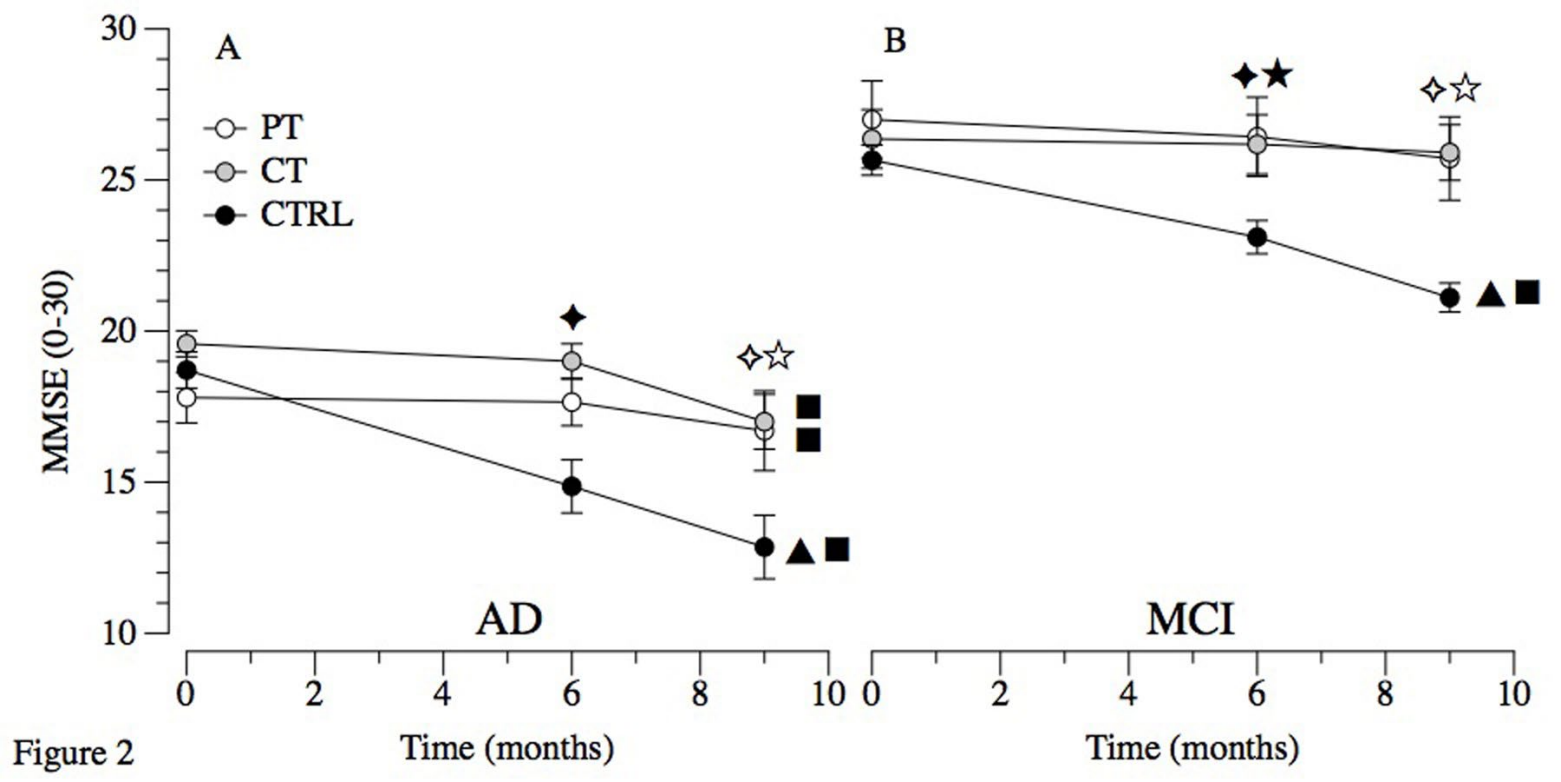
**Primary outcome in MCI and AD.** Abbreviations: MCI: Mild Cognitive Impairment; AD: Alzheimer’s Disease; PT: Physical Treatment group; CT: Cognitive Treatment group; CTRL: Control Group. Within-group comparison significant results (p ≤ 0.05): ▲ T0-T1; ■ T0-T2. Between-groups significant results (p ≤ 0.05): ★ T1 PT vs T1 CTRL; ✦ T1 CT vs T1 CTRL; ☆ T2 PT vs T2 CTRL; ✧ T2 CT vs T2 CTRL.

Post-hoc tests revealed no difference between the three treatments’ groups at T1 and T2 both in patients with MCI and AD. However, in MCI amelioration in CTRL were found (T0- T1: *P*=.039; T0-T2: *P*<.001). In AD worsening in CTRL (T0-T1: *P*<.001; T0-T2: *P*<.001), and amelioration in CT (T0-T2: *P*<.001) were seen (see Figure 2).

### Secondary cognitive and behavioral outcomes in MCI and AD

Significant effects of the factors Time (_F2,162_= 11.444; *P*<.001), Treatment (F_2,81_= 4.077; *P*=.020) and Group (_F1,81_= 39.840; *P*<.001) and Time x Treatment (F_4,162_= 10.887; *P*<.001) and Time x Group (F_2,162_= 5.277; *P*=.006) interactions on FAB. Post-hoc comparisons revealed no significant results in MCI, but in AD a significant difference between CTRL and CT in T2 (*P*=.041). Moreover, in AD a worsening of CTRL in time (T0-T1: *P*<.000; T0-T2: *P*<.000) was found.

Effects of the factors Time (F_2,162_= 29.885; *P*<.001) and Group (F_1,81_= 38.598; *P*<.001) and the Time X Treatment (F_4,162_= 5.032; *P*<.001) and Time X Treatment X Group (F_4,162_= 2.575; *P*=.039) interactions were found on IADL. Post-hoc did not reveal any difference at T1 and T2 between the three treatments’ groups in patients with MCI and in patients with AD. We found a worsening of CTRL between T0 and T2 (*P*<.001) in MCI, and differences from T0 to T2 in CT (*P*<.001) and CTRL (*P*<.001) in AD.

Significant effects of Time (F2,162= 18.425; *P*<.001), Treatment (F_2,81_= 18.204; *P*<.001), Group (F_1,81_= 15.255; *P*<.001) and Time X Treatment (F_4,162_= 21.339; *P*<.001) and Treatment X Group (F_2,81_= 6.605; *P*=.002) interactions on NPI. Post-hoc showed difference between groups at T1 (PT vs. CTRL: *P*<.001; CT vs. CTRL: *P*<.001) and at T2 (PT vs. CTRL: *P*<.001; CT vs. CTRL: *P*<.001) in AD. Moreover, we found changes from T0 to T2 in CTRL (*P*<.001) in MCI and worsening of CTRL across time (T0-T1: *P*=.001; T0-T2: *P*<.001) in AD (Table 2).

**Table 2:** Secondary cognitive and behavioral outcomes in MCI and AD.

	** Treatment**	**Groups**	**T0**	**T1**	**T2**	Within-group comparison (Time)	Between-groups comparison (Treatment)
							
**FAB** **(0-18)**	PT	MCI AD	12.9±2.5 8.9±2.4	13.4±3.6 9.9±2.4	12.7±3.5 8.5±2.8		
	CT	MCI AD	11.7±2 8.6±1.9	12.8±2.7 9.1±2.3	12.7±3.1 7.7±2.9		AD: ✧
	CTRL	MCI AD	11.7±3 10.3±2.6	10.2±3.6 7.3±2.9	9.8±3.4 5.3±2.8	▲ ■	
							
**IADL** **(0-100%)**	PT	MCI AD	88.2±23.6 56.2±35.1	86.4±22.1 50.5±32.9	81.8±21.2 39.2±31.2		
	CT	MCI AD	84.1±19.8 58.5±28.8	89.5±18.8 54.1±29.8	86.1±19.6 38.2±26.6	■	
	CTRL	MCI AD	84.4±25.9 48.9±23.4	73.1±34.3 34±25.5	56.4±33.8 21.1±18.3	■ ■	
							
**NPI** **(1-144)**	PT	MCI AD	11.7±9.1 12.7±8.7	7±4.2 9.5±6.8	9.9±5.7 11±5.4		
	CT	MCI AD	10.7±7.3 13.6±8.9	6±4.9 9.6±7.1	11.4±7.9 13.7±10.4		AD: ★✦☆✧
	CTRL	MCI AD	6.2±2.9 16.1±8.8	13.8±11.2 29.7±9.7	20.9±17.9 40±11.3	■ ▲ ■	

Data are given as mean ± standard deviation. Abbreviations: PT: Physical Treatment group; CT: Cognitive Treatment group; CTRL: Control group; FAB: Frontal Assessment Battery; IADL: Instrumental Activity of Daily Living; NPI: Neuropsychiatric Inventory. T0: Pre-Treatment assessment; T1: Post-Treatment assessment; T2: Follow-p assessment.

Within-group (Time) comparison significant results (p ≤ 0.05): ▲ T0-T1; ■ T0-T2.

Between-groups (Treatment) significant results (p ≤ 0.05): ★ T1 PT vs T1 CTRL; ✦ T1 CT vs T1 CTRL; ☆ T2 PT vs T2 CTRL; ✧ T2 CT vs T2 CTRL.

### Secondary cognitive outcomes specific for MCI

Significant effects of Time (F_2,48_= 7.33; *P*=.001), Treatment (F_2,24_= 5.286; *P*=.012) and Time X Treatment interaction (F_4,48_= 5.715; *P*<.001) on TMT-A. Post-hoc showed differences at T1 (PT vs. CTRL: *P*=.014; CT vs. CTRL: *P*=.040), and T2 (PT vs. CTRL: *P*=.001; CT vs. CTRL: *P*<.001). A worsening of CTRL across time was found (T0-T1: *P*=.006; T0-T2: *P*<.001).

In TMT-B, effects of Time (F_2,48_= 12.46; *P*<.001), Treatment (F_2,24_= 8.46; *P*=.001) and Time x Treatment (F_4,48_= 11.93; *P*<.001) interaction were found. Post-hoc showed differences at T1 between PT and CTRL (*P*=.002), and CT and CTRL (*P*<.001), both confirmed at T2 (*P*<.001). A worsening of CTRL was found between T0 and T1 (*P*<.001) and T0 and T2 (*P*<.001).

Effects of Time (F_2,48_= 16.88; *P*<.001), Treatment (F_2,24_= 3.434; *P*=.048) and Time X Treatment interaction (F_4,48_= 10.06; *P*<.001) on RBMT were found. Post-hoc tests showed differences at T1 (PT vs. CTRL: *P*=.022; CT vs. CTRL: *P*=.006) and T2 (PT vs. CTRL: *P*=.017; CT vs. CTRL: *P*=.028) and changes from T0 to T1 in all treatments’ groups (PT: *P*=.019; CT: *P*<.001; CTRL: *P*=.006), and from T0 to T2 in CTRL (*P*<.001) (see Table 3).

**Table 3:** Secondary cognitive outcomes specific for MCI.

	**Treatment**	**T0**	**T1**	**T2**		Between-groups comparison (Treatment)
						
**TMT-A (sec.)**	PT	95.6±15	82±12.2	94±17.3		★✦☆✧
	CT	87.6±28.2	97±47.1	97.1±36.3		
	CTRL	111.8±64.9	149.1±68.8	180.7±62.6	▲ ■	
						
**TMT-B (sec.)**	PT	209.1±48.7	190.1±30.6	195.7±39.8		★✦☆✧
	CT	193.9±56.5	173.1±53.3	215.2±76.5		
	CTRL	233±67.2	297.3±71	331.4±54.7	▲ ■	
						
**RBMT (0-212)**	PT	79±29.1	93.6±35.6	81.7±37	▲	★✦☆✧
	CT	77±30.3	95.1±31.4	74.5±36.2	▲	
	CTRL	66.2±22.3	51.6±25.4	38.3±21.1	▲ ■	

Data are given ad mean ± standard deviation. Abbreviations: PT: Physical Treatment group; CT: Cognitive Treatment group; CTRL: Control group. TMT: Trail Making Test; RBMT: Rivermead Behavioral Memory Test; FAB: Frontal Assessment Battery; IADL: Instrumental Activity of Daily Living; NPI: Neuropsychiatric Inventory. T0: Pre-Treatment assessment; T1: Post-Treatment assessment; T2: Follow-p assessment.

Within-group comparison significant results (p ≤ 0.05): ▲ T0-T1; ■ T0-T2.

Between-groups significant results (p ≤ 0.05): ★ T1 PT vs T1 CTRL; ✦ T1 CT vs T1 CTRL; ☆ T2 PT vs T2 CTRL; ✧ T2 CT vs T2 CTRL

### Secondary cognitive outcomes specific for AD

Effects of Time (F_2,114_= 30.81; *P*<.001) and Time X Treatment interaction (F_4,114_= 23.93; *P*<.001) were found on DCT. Within-group comparisons showed changes from T0 to T1 in PT (*P*=.002) and CTRL (*P*<.001), and from T0 and T2 in CTRL (*P*<.001).

Effects of the factors Time (F_2,114_= 49.05; *P*<.001) and the Time X Treatment interaction (F_4,114_= 15.48; *P*<.001) were found on ADAS-Cog, with changes in PT (T0-T1: *P*=.037; T0-T2: *P*=.005) and in CTRL (T0 to T1: *P*<.001; T0 to T2: *P*<.001).

Post-hoc did not show any difference at T1 and T2 between the three treatments’ groups both in DCT and in ADAS-Cog (Table 4).

**Table 4:** Secondary cognitive outcomes specific for AD.

						
	**Treatment**	**T0**	**T1**	**T2**	Within- group comparison (Time)	Between- groups comparison (Treatment)
						
**DCT (0-60)**	PT	25.5±11.4	29.5±10.8	25.0±10.6	▲	
	CT	23.9±9.3	25.5±7.8	22.9±10.2		
	CTRL	33.3±10.8	25.3±12.2	20.0±11.4	▲ ■	
						
**ADAS-Cog** **(0-70)**	PT	33.3±17.9	30.1±16.1	37.2±17.9	▲ ■	
	CT	27.1±7.6	25.5±7.5	30.1±9.2		
	CTRL	25.9±9.5	34±9.3	38.7±10.8	▲ ■	
						

Data are given as mean ± standard deviation. Abbreviations: PT: Physical Treatment group; CT: Cognitive Treatment group; CTRL: Control group; DCT: Digit Cancellation Test, ADAS-Cog: Cognitive section of the Alzheimer’s Disease Assessment Scale; FAB: Frontal Assessment Battery; IADL: Instrumental Activity of Daily Living; NPI: Neuropsychiatric Inventory. T0: Pre-Treatment assessment; T1: Post-Treatment assessment; T2: Follow-p assessment.

Within-group comparison significant results (p ≤ 0.05): ▲ T0-T1; ■ T0-T2.

Between-groups significant results (p ≤ 0.05): ★ T1 PT vs T1 CTRL; ✦ T1 CT vs T1 CTRL; ☆ T2 PT vs T2 CTRL; ✧ T2 CT vs T2 CTRL.

### Exercise capacity and cardiovascular risk factors in MCI and AD

Effects of Time (F_2,162_= 5.526; *P*=.004) and Time X Treatment (F_4,162_= 9.673; *P*=.040) and Time X Treatment X Group (F_4,162_= 2.560; *P*=.040) interactions on BMI were seen. No between-groups differences were found in the post-hoc analysis. However, they indicated anBMI increased for CTRL in MCI (T0-T2: *P*=.008) and changes from T0 to T1 (*P*=.011) for PT in AD group.

Effects of the factors Time (F_2,162_= 18.663, *P*<.001), Treatment (F_2,81_= 5.322, *P*=.006) and Group (F_1,81_= 10.806; *P*=.001) and Time X Treatment interaction (F_4,162_= 15.487; *P*<.001) were found on 6MWT. Post-hoc analysis did not show any difference between the three treatments’ groups both in AD and in MCI. We showed changes in CTRL both in MCI and in AD from T0 to T2 (*P*=.004 and *P*<.001 respectively) and from T0 to T1 for CTRL in AD (*P*<.001).

Effects of the factors Time (F_2,162_= 22.53, *P*<.001), Treatment (F_2,81_= 13.10, *P*<.001) and the Time X Treatment interaction (F_4,162_= 26.76; *P*<.001) on systolic blood pressure were found. Post-hoc showed differences in MCI at T1 (PT vs. CTRL: *P*=.008) and in AD at T1 (PT vs. CTRL: *P*=.001) and at T2 (PT vs. CTRL: *P*=.025, CT vs. CTRL: *P*=.016). Moreover, changes in PT and in CTRL were found in MCI (PT, T0-T1: *P*=.003; CTRL, T0-T1: *P*=.041, T0-T2: *P*=.001) and in AD (PT, T0-T1: *P*<.001; CTRL, T0-T1: *P*=.002, T0-T2: *P*<.001).

Effects of the factors Time (F_2,162_= 12.41, *P*<.001), Treatment (F_4,81_= 4.63, *P*=.012) and the Time X Treatment (F_4,162_= 24.70, *P*<.001) and Time X Treatment X Group (F_4,162_= 2.69, *P*=.033) interaction on diastolic blood pressure were seen. Post-hoc showed differences between PT and CTRL at T1 both in MCI and in AD (*P*=.002, *P*=.002). A worsening in CTRL (T0-T1: *P*<.001, *P*=.001; T0-T2: *P*<.001, *P*<.001) were found both in MCI and AD. An improvement was found for PT in AD (T0-T1: *P*<.001).

Effects of Time (F_2,162_= 9.520; *P*<.001), Group (F_1,81_= 14.985; *P*<.001) and Time X Treatment (F_4,162_= 12.581; *P*<.001) and Time X Group (F_2,162_= 3.978; *P*=.020) interactions were found in glucose blood level. No between-groups differences were shown in the post-hoc analysis, but an improvement was found for PT in MCI and AD from T0 to T1 (*P*<.001, *P*=.010).

Effects of the factors Treatment (F_2,81_= 3.261; *P*=.043) and Group (F_1,81_= 16.672; *P*<.001) on total cholesterol, with no between-groups changes in the post-hoc analysis, but significant difference for CTRL in AD between T0 and T2 (*P*=.032) were found.

For HDL, only the Time X Treatment (F_4,162_= 6.412, *P*<.001) and Time X Treatment X Group (F_4,162_= 7.526, *P*<.001) interactions were significant, with neither between nor within-groups effects in the post-hoc analysis.

Effect of Time (F_2,162_= 5.428, *P*=.005), Treatment (F_1,81_= 36.252, *P*<.001) and Time X Treatment (F_4,162_= 2.966; *P*=.021), Time X Group (F_2,162_= 16.230; *P*<.001) and Time X Treatment X Group (F_4,162_= 6.955; *P*<.001) interactions on LDL were found. Post-hoc analysis showed no between-groups differences, but changes in PT both in MCI and in AD (T0-T1: *P*=.015, *P*<.001).

For triglycerides, an effect of Time (F_2,162_= 10.201; *P*<.001) and Time X Treatment interaction (F_2,162_= 6.771; *P*<.001) were found. Post-hoc analysis did not find any difference between the three treatments’ groups, but a difference for PT in AD between T0 and T1 (*P*<.001; Table 5).

**Table 5:** Exercise capacity and cardiovascular risk factors in MCI and AD.

	**Treatment**	**Groups**	**T0**	**T1**	**T2**	Within-group comparison (Time)	Between-groups comparison (Treatment)
							
**BMI (kg/m^2^)**	PT	MCI AD	28.5±4.8 25.6±3.4	27.1±4.4 24.5±2.8	27.1±4.4 25.6±3.17	▲	
	CT	MCI AD	26.2±5.1 25.8±5.5	25.8±5 25.6±5.3	26±5.1 26.2±5.5		
	CTRL	MCI AD	27±3.2 26.9±3.1	28±3.5 27.4±3.3	28.7±3.1 27.6±3.4	■	
							
**6MWT (m)**	PT	MCI AD	391.9±57.1 323.1±115.4	447.9±73.8 347.6±94.4	398.3±69.8 334.1±116.3		
	CT	MCI AD	440.1±95.4 336±109.2	399.7±90.9 318.2±106.3	395.9±68.9 317.3±105.5		
	CTRL	MCI AD	352.8±55.4 342.5±40.9	314.6±44.4 271±73.3	285.4±29.3 253.1±74.2	■ ▲■	
							
**SYS (mmHg)**	PT	MCI AD	130.1±6.1 129.2±5	125.9±3 124.4±4.2	130±4.9 129±4.6	▲ ▲	MCI: ★ AD: ★☆✧
	CT	MCI AD	130.6±3.6 128.7±6.2	130.2±4.1 128.7±6.3	131.2±3 128.7±6.4		
	CTRL	MCI AD	135.2±11.5 132.3±6	138.44±9.5 134.8±5.4	139.3±9.4 136.2±5	▲ ■ ▲ ■	
							
**DIA (mmHg)**	PT	MCI AD	87.9±7.1 87.7±3.6	84.4±5.1 84.6±2.4	88.3±5.6 87.6±2.6	▲	
	CT	MCI AD	88±3.7 87.1±3.5	87.1±5.1 87.8±3.1	87.4±4.2 88±3.3		MCI: ★ AD: ★
	CTRL	MCI AD	86.9±2.3 86.6±2.1	91.9±3.1 89.2±1.2	91.7±2.1 89.4±1	▲ ■ ▲ ■	
							
**GLUCOSE (mg/dL)**	PT	MCI AD	119.6±24.2 99.3±8.6	98.43±5 91±10.4	112.3±16.9 95.6±9	▲ ▲	
	CT	MCI AD	106±19.1 98.2±12.6	105.3±20.9 97.9±13.2	111.4±19 97.6±13.1		
	CTRL	MCI AD	103±12.8 96.3±13.2	107.7±9.6 98.9±12.1	109±9.5 98.2±11		
							
**TOTAL CHOLESTEROL (mg/dL)**	PT	MCI AD	167.7±16.9 207.3±34.2	135.7±9.5 189±36.3	161±11.3 20.5±27.6		
	CT	MCI AD	174.9±28.2 200.8±24.3	177.5±37.5 196.7±23.3	171.2±23.1 198.5±18.6		
	CTRL	MCI AD	182.9±16.4 196.4±28.2	189.4±13.9 206.8±29.9	191.2±15.1 250.6±154.9	■	
							
	PT	MCI AD	63±13 50.2±9.7	68.7±10.2 55±12.2	57.4±16.8 52.1±11.9		
**HDL (mg/dL)**	CT	MCI AD	58.8±21.4 59.3±15.2	57.8±15.7 54±14.2	52.8±10 58.2±12.3		
	CTRL	MCI AD	57.6±9.3 55.6±10.7	53.6±11.5 54.2±9.8	63.3±8.1 51.6±8.5		
							
	PT	MCI AD	90.1±14.1 124.8±18.7	105.9±14 112.2±16.6	89.7±16.3 123±20.1	▲ ▲	
**LDL (mg/dL)**	CT	MCI AD	94.4±12.3 120.7±21	100.7±12.7 118.8±20.2	98±13 120.4±17.7		
	CTRL	MCI AD	90.8±6.4 119.7±23.3	100.1±5.4 125.3±22.9	102.3±4.7 125.7±21.4		
							
	PT	MCI AD	125.4±11.7 129.2±41	111.6±16.4 111.8±36.3	129.7±18.7 126.1±37.6	▲	
**TRIGLYCERIDES** **(mg/dL)**	CT	MCI AD	115.55±12.6 118.8±37.8	115.6±10.5 125.4±37.7	119±12.5 127.5±36.4		
	CTRL	MCI AD	114.2±18.3 124.2±22.9	118.1±17.6 128.9±25.1	123.3±14.3 132±24.4		

Data are given as mean ± standard deviation. Abbreviations: PT: Physical Treatment group; CT: Cognitive Treatment group; CTRL: Control group. BMI: Body Mass Index; 6MWT: 6-Minute Walking Test; SYS: Systolic blood pressure; DIA: Diastolic blood pressure; HDL: High-Density Lipoprotein, LDL: Low-Density Lipoprotein. T0: Pre-Treatment assessment; T1: Post-Treatment assessment; T2: Follow-p assessment.

Within-group comparison significant results (p ≤ 0.05): ▲ T0-T1; ■ T0-T2.

Between-groups significant results (p ≤ 0.05): ★ T1 PT vs T1 CTRL; ✦ T1 CT vs T1 CTRL; ☆ T2 PT vs T2 CTRL; ✧ T2 CT vs T2 CTRL

## DISCUSSION

The aim of this RCT was to evaluate the effects of CT and PT on the progression of the cognitive deficits in MCI and AD. In agreement with our hypothesis, the natural progression of the cognitive symptoms for both MCI and AD was mitigated by CT and PT. Specifically, our results confirm the hypothesis that both treatments are successful in slowing down the usual worsening of cognitive symptoms in patients with MCI and AD. Also, secondary outcomes suggest that both treatments have positive effects on memory and attention abilities in patients with MCI. It is important to note that a general amelioration of the cardiovascular risk factors and exercise capacity were retrieved in both MCI and AD after PT. Long term effects of both CT and PT seem to persist after the end of the treatments. Although between groups differences at T1 and T2 were generally not found, results indicate that MCI retain better than AD the achieved adaptations, suggesting that the latter may better benefit from a constant rather than a periodic treatment. Overall the results of this study suggest that PT and CT have similar effectiveness in several cognitive domains and can be incorporated among the non-pharmacological treatments for patients with MCI and AD.

### Impact of CT and PT on global cognitive impairment in patients with MCI

The results of this study indicate that the overall cognitive worsening (measured with MMSE) are reduced in patients with MCI undergoing CT and PT. Indeed, this study demonstrates a significant difference for both experimental treatments in comparison to the control group (Figure 2, Panel A). Interestingly, these positive effects are persistent for both CT and PT leading to long-term effects significantly detectable 3 months after the treatment ended. As expected, and previously reported by our group [16] CTRL underwent to a significant decline. The rapid decline in cognitive functioning is commonly reported in the literature that reported a loss of 3 or more points on the MMSE score in 6 months [12,17]. The effects of cognitive treatments in postponing cognitive decline in persons with MCI is also confirmed in a recent meta-analysis that showed memory and multidomain-lifestyle interventions to facilitate partial activation of compensatory scaffolding and neuroplasticity [18]. The effectiveness of PT were confirmed in reviews and meta-analysis [5,12,14,19,20] that showed PT, in particular aerobic exercise, to improves global cognitive scores [21–23], with a moderate but significant effect on memory [5] and executive control processes such as planning, scheduling, dealing with ambiguity, working memory and multitasking [24]. Overall, our results are highly relevant because for the first time the efficacy of a PT has been compared with a CT, and the potential integration of these successful approaches in the standard clinical scenario likely expand the possible treatments.

### Impact of CT and PT on global cognitive impairment in patients with AD

The results of this study indicate that both CT and PT preserved the cognitive status in AD during the six months of treatment. Unfortunately, both groups but in particular CT exhibited a severe drop in the cognitive performance 3 months after the training (Figure 2, Panel B). This lack of long-term effects is probably due to the more severe cognitive and physical impairments of these patients, which may require continuous treatments. As expected, the global cognitive status of the CTRL group progressively worsened.

Our data are in agreement with the positive effect of CT on general cognition in AD [15]. Moreover, the positive effects of PT in our RCT are in line with several recent studies in AD [25–30]. As previously reported by our group [28,31], it is possible to stabilize the progressive cognitive dysfunctions in nursing home residents with AD through a specific moderate intensity endurance and resistance training. These data suggest that the practice of regular physical activity might contribute to slower cognitive decline. However, ~57% of previous studies used the MMSE as the only cognitive outcome measure [32], and this may not be sensitive enough to change because it does not explore in depth any cognitive domain, and in particular the memory deficits associated with AD. The use of other cognitive outcomes in this study further supports the effectiveness of CT and PT.

### Impact of CT and PT on specific cognitive domains in patients with MCI

In patients with MCI we observed that 6 months of CT or PT improved memory compared with CTRL group. Furthermore, both CT and PT have an impact on selective attention, shifting ability and executive functions.

The effects of CT on mental flexibility, memory, executive function, processing speed, attention, and fluid intelligence was demonstrated in a previous RCT [25] and systematic review [33].

Exercise to prevent dementia and delay cognitive decline have gained considerable attention in recent years [34]. In particular, several studies have demonstrated that PT can impact attention [26,35,36] and executive functions [27,35]. However, conflicting results are present in the literature on delayed recall [28,36]. Nevertheless, previous studies reported relatively short duration of PT (6 weeks - 3 months), and the compliance was rarely reported. These two variables may explain less efficacy of PT on memory in MCI. Our data suggest that PT or CT may alter the trajectory of specific cognitive domains decline in MCI. These results confirm the efficacy of CT and PT on cognitive decline in MCI and corroborate the need to add these strategies to pharmacological treatment.

### Impact of CT and PT on specific cognitive domains in patients with AD

CT and PT had an effect on some cognitive domains in AD, but in comparison to MCI these effects dropped quickly after the end of treatment. The effect of PT on attention and global cognition after the treatment is in keeping with previous studies [29]. Overall the results obtained in AD and MCI converge towards a possible overlap of the effects of the two treatments. Indeed, the effects of treatments seem to vanish after 3 months of inactivity, suggesting the need of a constant training.

### Impact of CT and PT on cardiovascular risk factors in patients with MCI

In patients with MCI we observed that 6 months of PT showed significant ameliorations of BMI, 6MWT, systolic and diastolic blood pressure, glucose, cholesterol and triglycerides. On the contrary, these parameters were not changed after CT and worsened in the CTRL group.

Strong evidence supports the notion that cardiovascular disease risk factors, such as hypertension, hypercholesterolemia, and glucose intolerance, contribute to the onset, development and exacerbation of dementia [4,31,37] and many studies suggest the opportunity of using physical exercise for both, primary and secondary AD prevention.

### Impact of CT and PT on cardiovascular disease risk factors in patients with AD

In patients with AD, the analysis of cardiovascular risk factors at T1 revealed significant amelioration in BMI, systolic and diastolic blood pressure, glucose and triglycerides in PT group. Indeed physical activity is known to be the most potent long-term vaso-protective non-pharmacological treatment and has a strong impact on many of those factors [31,38,39], influencing the threshold of manifestation of AD by way of strengthening vascular plasticity [38]. Moreover, higher cardiorespiratory fitness is associated with a diminution of Aβ related effects on cognition, suggesting that exercise might play an important role in AD [40].

Exercise-induced effects on cardiovascular system might be largely explained by a variety of vascular and cardiac molecular mechanisms that provide a protective environment in cardiovascular system, this beneficial effect can be extended to cerebral vasculature as well [38].

However, these cardiovascular risk factors improvements were not maintained 3 months after the end of PT, suggesting, the need of a constant training for patients with AD. As expected, these cardiovascular disease risk factors were not affected by the CT, who underwent a worsening of triglycerides.

### Responsible pathways underlying the effectiveness of the two treatments

The positive cognitive outcome retrieved in this RCT are likely induced by different physiological effects induced by CT or PT. For instance, it is well established that regular exercise lowers the blood pressure and lipids, preventing metabolic syndrome and having positive effects on inflammatory markers and endothelial functions, recognized risk factors for AD. Moreover, the current literature demonstrates that six months of aerobic training in 70 to 80 year-old community-dwelling women with probable MCI, may increase hippocampal volume by increasing levels of BDNF, which stimulate neurogenesis and increase the complexity of the dendritic network. Erickson et al. [64] found that one year of aerobic exercise in late adulthood is sufficient to enhancing hippocampus volume. This volume enhancement translates to improved memory function. Therefore, PT may be neuroprotective and starting an exercise regimen later in life is not futile for either enhancing cognition or augmenting brain volume [64]. Moreover, chronic aerobic exercise improves regional cerebral blood flow in various relevant brain structures, primarily in hippocampus, in response to cognitive tasks along with better task performance [41].

On the other side, CT in MCI and AD seems to improve cognitive reserve. The current literature reported that this resulted in significantly slower decline of brain metabolism, especially in left anterior temporal pole and anterior cingulate cortex [42].

## Limitations of Study

A limitation of the current study was the relatively small sample size, which may have influenced the differences induced by the training adopted in the participants. However, due to the complexity of the study and the limited availability of the participants eligible for the present investigation, the sample size was small. Further limitations were the mixed gender of the sample, and the potential effects of comorbidities. Another limitation of the study is the use of the RPE during the PT. It is known that the effort perceived by demented individuals might be altered by the disease itself, thus using RPE scale may give wrong feedback if this is used as a unique method to monitor exercise intensity. However, we used the RPE scale together with the HR monitor during every exercise session to understand the state of the participants and to have an instantaneous feedback about the effort feeling while exercising.

## CONCLUSION

This study confirms the positive effect of CT and PT on cognitive impairment in MCI and AD. The results contribute to the growing body of literature that indicates the potentially beneficial relationship between physical exercise and cognition, this is the first study demonstrating that CT was not superior to PT.

## METHODS

### Trial design

A single-blind randomized controlled trial (RCT) comparing the effects of CT with PT on cognitive performance was performed. The examiner was blinded to group assignment (allocation ratio 1:1). The study was carried out in accordance with the Helsinki Declaration and approved by the ethics committee of the University Hospital Verona, Italy (Protocol CE 2389; ClinicalTrials.gov Identifier is NCT03034746). The study was reported in accordance with the CONSORT guidelines.

### Participants

Outpatients with MCI and AD were recruited from the Department of Neuroscience, Biomedicine and Movement Sciences (University of Verona) and Mons. Mazzali Geriatric Institute between January 2014 and February 2016.

Inclusion criteria were (a) aged 65-90 years; (b) clinical diagnosis of MCI due to AD and probable AD dementia, established according to the National Institute on Aging-Alzheimer's Association diagnostic guideline for MCI due to AD and AD [43,44], (c) Performance Oriented Mobility Assessment-POMA>19) [45].

Exclusion criteria were: modifications of medications during the last 3 months, a history of depression or psychosis, alcohol or drug abuse, other neurological, cardiac, orthopedic, or respiratory pathology (e.g., chronic obstructive pulmonary disease).

After a first evaluation, patients were randomized assigned to CT, PT or control group (CTRL). The flow chart of the study is reported in Figure 1.

Patients and their relatives were informed about the experimental nature of the study and gave their written informed consent.

### Interventions

Each patient underwent a group treatment 90-minute/day, three days/week for 72 treatment sessions. Each group included 7-8 patients with the same degree of cognitive decline. During the study patients were not allowed other types of PTs or CTs.

CTRL group received the standard pharmacological treatment. PT and CT groups kept previous pharmacological treatment. During the study, drug therapies were unchanged.

### Cognitive treatment

CT, conducted by two neuropsychologists (ratio 2:5), was adjusted according to the severity of the cognitive decline observed, replicating or adapting two programs present in literature [32,46]. For patients with MCI, CT aimed to reduce the impaired skills, acquire compensatory strategies using external aids, and use ecological materials such as the reconstruction of scenarios related to daily life situations. The intervention program has been configured as a cognitive rehabilitation and mainly memory rehabilitation: the participants were trained in practicing restorative and compensatory mnemonic techniques, such as visual imagery, face-name association, calendar, notes and prompts. In patients with AD, CT was based upon the stimulation (and not rehabilitation) of residual cognitive skills. Each session began with an introduction of each subject to the other members of the group, aiming to provide continuity and orientation by beginning all sessions in the same way. After that, oral and paper-pencil exercises of specific cognitive functions were proposed. The session also included activation of everyday life activities, leisure activities and topics of common interest (e.g. music and food), taking into account the group’s cognitive capabilities. These exercises aimed to the natural process of reminiscence, but they also focused on the present situation, having an impact on social interaction and mood. Multisensory stimulation was introduced.

### Physical treatment

PT, conducted by two kinesiologists (ratio 2:5), included moderate intensity endurance and resistance training. Sessions started with 15 minutes of warm-up which included active joint mobilization and walking on treadmill at preferred speed. Then, patients performed 45-minute of endurance exercises divided in: 15-minute of cycling on cycle ergometer, 15-minute of walking on a treadmill, 15-minute of arm cranking on a specific ergometer with a random order. The 70% of maximal heart rate was calculated using the Karvonen formula [47] (220-age in years) because no specific equation are validated in patients with dementia. For participants taking beta-blockers the 65% of (220-age) was considered, as suggested by Carvalho et al. [48]. Work load intensity was increased, if it was possible, by 5% every 6 weeks and was monitored by HR monitor belt and by the Rating of Perceived Exertion scale (RPE) [49]. The RPE scale was not used to set the intensity, which was based on HR of the participant during each aerobic exercise, but it was used as an extra tool to monitor how patients perceived the effort during the aerobic training section. Although participants were not completely naïf to aerobic exercise, exercising at a certain intensity for some minutes was not so easy for them, especially at the beginning of the study. The RPES scale was useful to monitor the global effort experienced by the patients during the training. Furthermore, patients started with a low intensity aerobic training in the first PT sessions, aiming to reach the 70% intensity in 2 weeks. This allowed to set the right intensity for all the exercise included in this training section, in particular for the arm cracking device which may be more challenging than other training equipment. All the participants reached the required intensity within 2 weeks.

Subsequently, patients performed 3 sets of 12 reps of resistance exercises at 85% of 1 repetition maximum (1RM), estimated with the Brzicky methods, for isotonic ergometers including chest-press, lat-machine, leg-press [50]. Selected patients were all naïf to resistance training and due to the short familiarization (1 day) with exercise devices, the estimate of the one repetition maximum was likely underestimated. Therefore, during the first week of PT we asked the participants to perform as many repetitions as possible with the 85% of the estimated 1RM. Furthermore, as soon as participants were able to perform the 12 repetitions easily (that means they were able to execute more than 12 repetitions) the workload was increased by 5%.

PT ended with stretching exercises for all the muscles involved in the training. The kinesiologists motivated the participants and gave patients time to perform the exercise as a whole.

### Outcome measures

Primary and secondary outcome measures were measured by the same blinded examiners before (T0), immediately after treatment (T1), and at three-months follow-up (T2). The cognitive assessment was carried out in one day, specifically in 2 hours, in the order in which they are mentioned above. The physical function assessment was carried out the next day.

### *Primary outcome*

The primary outcome was the *Mini Mental State Examination (MMSE)* to assess the global cognitive impairment [51]. Although the MMSE is a screening test, it has been used as primary outcome measure in studies on AD [52,53].

### *Secondary outcomes*

#### Secondary outcomes for MCI:

*Trail Making Test* to evaluate the attention ability, in particular selective attention, psychomotor speed and sequencing skills (TMT-A) and the ability to switch attention between two rules or tasks (TMT-B). The time taken to complete the trails was recorded [54].

*Rivermead Behavioral Memory Test (RBMT)*, an ecological memory battery resembling everyday tasks, with the aim to measure daily memory function. The RBMT-3 consists of ten subtests (Names, Belongings, Appointments, Picture Recognition, Story, Faces, Route, Message, Orientation, Novel Task) and has two parallel versions for monitoring changes over time [55].

#### Secondary outcomes for AD:

*Digit Cancellation Test (DCT)* to assess visual-selective attention. Three matrices are shown to the subject and the patients has to cross the target stimuli between distractor stimuli [56].

*Cognitive section of the Alzheimer’s Disease Assessment Scale (ADAS-Cog)* to assess the global cognitive decline investigating skills in 9 functional sub-test (i.e. comprehension, memory and execution of orders) and 2 memory sub-tests (words recall and recognition) [57].

#### Secondary outcomes for MCI and AD:

*Frontal Assessment Battery (FAB)* it is a short cognitive and behavioural six-subtest battery that assess executive functions (similarities: participants have to identify the link between two objects from the same semantic category and it explores conceptualisation; phonological verbal fluency: participants have to produce in a minute as many words as they can beginning with the letter “S” and it explores mental flexibility; motor series: participants have to perform Luria’s “fist-edge-palm” series six times consecutively and this task explores motor programming; conflicting instruction: participants have to provide an opposite response to examiner’s alternating signal and it explores sensitivity to interference, go-no go task: it is used the same alternating signals of the previous task but here participants have to provide different responses and this task explores inhibitory control; prehension behaviour: the examiner touches both participant’s palms and this explores the spontaneous tendency to adhere to the environment and environmental autonomy) [58].

*Instrumental Activities of Daily Living (IADL)* to assess the independence of patient in some instrumental activities of daily living (i.e. use of the telephone, shopping, food preparation) (Range:0-5/8; higher score indicates better autonomy) [59].

*Neuropsychiatric Inventory (NPI)* to evaluate the presence, frequency and severity of behavioral disorders [60].

*Body mass index (BMI)* to measure general body composition. Fasted body mass and height were measured in the morning with a professional mechanical scale fitted with a stadiometer (Seca mod. 713; III-M; Seca Medical Scales and Measuring Systems, Birmingham, UK). BMI was than calculated as body mass relative to squared height.

*Six-Minute Walking Test (6MWT)* to measure the maximum distance that a person can walk over 6 min and it is commonly used as an assessment of exercise capacity. The participants were instructed to walk from one end of a 30-meter course to the other and back again as many times as possible in 6 min, under the supervision of a kinesiologist. After each minute, participants were informed of the time elapsed and were given standardized encouragement. The distance (meters) covered in 6 minutes was recorded [61].

*Blood pressure:* One skilled physician measured blood pressure with standard auscultatory and mercury sphygmomanometer technique at about the same time of the day to minimize the effect of circadian rhythm on the measurement. The standard error of measurement of systolic blood pressure and diastolic blood pressure are ± 0.7 (mmHg), and ±1.1 (mmHg), respectively [62].

*Blood sample and analysis*: Venous peripheral blood (25 mL) was collected between 9:00 and 10:00 am in a fasted state and processed within 2 hours to obtain routine measurements of blood (Glucose, Total Cholesterol, High-Density Lipoprotein-HDL, Low-Density Lipoprotein-LDL, Triglycerides).

### Randomization and masking

After screening, participants were allocated to one of three arms according to a simple software-generated randomization scheme (www.randomization.com): (1) CT group, (2) PT group, and (3) CTRL group. The research team included “evaluators” and “treatment givers”. Evaluators were uninformed about group assignments, including physician and neuropsychologist who performed outcome measures. Treatment givers included neuropsychologists and kinesiologists who administered CT and PT, respectively.

### Statistical analysis

The sample size has been calculated based on the MMSE. Indeed, to obtain a significant effect size of 2 MMSE points [63], a sample size of 90 participants was chosen to guarantee a power of the study of 99% and a Type I error of 1%.

Statistical analysis was carried out using the PRISM statistical package, version 6 and STATISTICA package.

A one-way (1x3) analysis of variance (ANOVA) was applied to age, education, MMSE, and POMA between-groups to test the homogeneity of the groups before the study. A three-way (3x3x2) repeated-measure ANOVA (rm-ANOVA) was carried out on the primary outcome and on secondary outcomes that were explored both in MCI and AD, with “Time” as within-group factor, and “Treatment” (PT, CT, CTRL) and “Group” (MCI, AD) as between-group factors.

A two-way (3x3) rm-ANOVA, with “Time” as within-group factor, and “Treatment” (PT, CT, CTRL) as between-group factors was applied to secondary outcome measures tested in MCI and AD groups only.

In the presence of significant effects, a multiple comparisons tests with Bonferroni’s correction was performed. The familywise alpha level for significance was set at 0.05 (two-tails), with Bonferroni’s correction when needed, for all the analyses.
